# The impact of bilateral injuries on the pathophysiology and functional outcomes of volumetric muscle loss

**DOI:** 10.1038/s41536-022-00255-2

**Published:** 2022-10-15

**Authors:** Connor P. Dolan, Andrew R. Clark, Jessica M. Motherwell, Naveena B. Janakiram, Michael S. Valerio, Christopher L. Dearth, Stephen M. Goldman

**Affiliations:** 1 DoD-VA Extremity Trauma and Amputation Center of Excellence, Bethesda, MD USA; 2 Department of Surgery, Uniformed Services University of the Health Sciences and Walter Reed National Military Medical Center, Bethesda, MD USA

**Keywords:** Physiology, Preclinical research

## Abstract

Volumetric muscle loss (VML)—defined as the irrecoverable loss of skeletal muscle tissue with associated persistent functional deficits—is among the most common and highly debilitating combat-related extremity injuries. This is particularly true in cases of severe polytrauma wherein multiple extremities may be involved as a result of high energy wounding mechanisms. As such, significant investment and effort has been made toward developing a clinically viable intervention capable of restoring the form and function of the affected musculature. While these investigations conducted to date have varied with respect to the species, breed, and sex of the chosen pre-clinical in-vivo model system, the majority of these studies have been performed in unilateral injury models, an aspect which may not fully exemplify the clinical representation of the multiply injured patient. Furthermore, while various components of the basal pathophysiology of VML (e.g., fibrosis and inflammation) have been investigated, relatively little effort has focused on how the pathophysiology and efficacy of pro-regenerative technologies is altered when there are multiple VML injuries. Thus, the purpose of this study was two-fold: (1) to investigate if/how the pathophysiology of unilateral VML injuries differs from bilateral VML injuries and (2) to interrogate the effect of bilateral VML injuries on the efficacy of a well-characterized regenerative therapy, minced muscle autograft (MMG). In contrast to our hypothesis, we show that bilateral VML injuries exhibit a similar systemic inflammatory response and improved muscle functional recovery, compared to unilateral injured animals. Furthermore, MMG treatment was found to only be effective at promoting an increase in functional outcomes in unilateral VML injuries. The findings presented herein add to the growing knowledge base of the pathophysiology of VML, and, importantly, reiterate the importance of comprehensively characterizing preclinical models which are utilized for early-stage screening of putative therapies as they can directly influence the translational research pipeline.

## Introduction

Extremity injuries accounted for as many as 54% of all combat wounds sustained by injured U.S. Service members (SMs) in the Global War on Terror^1^. While significant advances in personnel protective measures (e.g., up-armored vehicles, body armor) and combat casualty care have greatly improved survival rates for the combat wounded^2^, the morbidity experienced by those SMs with traumatic extremity injuries has increased such that they require the longest average inpatient stays and experienced the greatest burden of repeated hospitalization of all the wounded in action. Despite this resource intensive care, these SMs still exhibited a 64% permanent disability rate^3,4^. Among the various extremity injury patterns contributing to this observed morbidity is volumetric muscle loss (VML), a debilitating condition characterized by a frank loss of skeletal muscle mass which results in chronic functional deficits^5^. VML presents pervasively with representation in ~50% of total combat-related extremity injuries, and the current standard of care is insufficient at restoring maximal muscle function and cosmesis. VML injuries progress down a natural, fibrotic repair pathway which results in the deposition of non-contractile fibrous tissue. Furthermore, architectural^6^, neural^7,8^ and oxidative^9^ pathology associated with the injury contributes to the continued degeneration of the functional capacity of the involved musculature over time. All of these factors contribute to make VML a prominent driver of disability among the wounded in action^10^. Exacerbating the prognosis of SMs with VML is the fact that high energy wounding mechanisms, which have been more prevalent in contemporary combat^11^, tend to simultaneously affect multiple body regions. As such, it is highly likely a large portion of SMs with VML are multiply injured, and thus have a high overall injury burden which is a known factor driving poor clinical outcomes^12,13^.

While there are examples of small^14–26^ and large^27–31^ animal studies using bilateral study designs, the majority of VML research interrogating the pathophysiology of VML or evaluating putative interventions for the treatment thereof have been conducted in translational models consisting of a single injury affecting one hindlimb of a quadruped species. Given that there is growing evidence on the systemic sequelae of VML, and that the likelihood of multiply injured VML patients within out the combat wounded SM population is high, there is an urgent need to understand the potential impact of increased injury burden on the wound healing environment of VML injuries and ultimately the efficacy of putative interventions under these conditions to better inform the continued translation of promising therapies and/or rehabilitation strategies. The objective of this study, therefore, was two-fold: (1) to investigate how the pathophysiology of unilateral VML injuries differed from bilateral VML injuries and (2) to interrogate the effect of bilateral injuries on the efficacy of a well characterized regenerative therapy, minced muscle autograft (MMG)^32–42^. It was hypothesized that bilateral VML-injured animals would exhibit worse outcomes by all measurements (muscle function, enhanced fibrosis, etc.) compared to unilateral VML injured animals, which would be mediated by a robust, deleterious, and prolonged systemic inflammatory response. Furthermore, we anticipated that increased systemic inflammation would also reduce the efficacy of MMG in bilaterally VML injured animals relative to their unilaterally injured counterparts.

## Results

### Bilateral VML injuries do not alter gross anatomy outcomes

We first investigated whether the number of VML injuries (i.e., unilateral or bilateral) with or without a regenerative treatment (i.e., No Repair or MMG) impacted body weights, and if defect weights were consistent across different experimental conditions (Table 1). Surgery weights were similar across experimental groups (*P* = 0.209, ANOVA) and no difference in defect weights was found between experimental groups (*P* = 0.609, ANOVA). No change was observed in body weight as a function of either number of injuries (*P* = 0.389, ANOVA) or treatment (*P* = 0.298, ANOVA).

**Table 1:** Animal, muscle, and defect weights.

	Unilateral (*n* = 7)		Unilateral + MMG (*n* = 8)		Bilateral (*n* = 8)		Bilateral + MMG (*n* = 9)		2-way ANOVA		
	Mean ± SEM								Main effects (*P* value)		
	VML	Sham	VML + MMG	Sham	VML	VML	VML + MMG	VML	Treatment	# of Injuries	Interaction
Surgery weight (g)	368.00 ± 5.44		364.25 ± 7.48		356.88 ± 3.80		369.22 ± 7.00		0.626	0.498	0.209
Sac weight (g)	434.43 ± 4.44		430.38 ± 10.67		414.25 ± 6.62		432.89 ± 8.95		0.298	0.389	0.184
Weight change (g)	66.43 ± 4.82		66.13 ± 6.73		57.38 ± 4.32		63.33 ± 3.59		0.243	0.574	0.534
TA weight (g)	0.53 ± 0.04^*^	0.77 ± 0.02	0.62 ± 0.01^*^	0.75 ± 0.02	0.58 ± 0.04	0.57 ± 0.04	0.69 ± 0.04^*^	0.59 ± 0.02	0.093	**0.004**	0.700
EDL (g)	0.19 ± 0.01	0.18 ± 0.01	0.17 ± 0.01	0.18 ± 0.01	0.16 ± 0.01	0.19 ± 0.01	0.16 ± 0.02	0.18 ± 0.01	0.100	0.211	0.417

	Mean ± SEM								Ordinary one-way ANOVA	
	VML	Sham	VML + MMG	Sham	VML	VML	VML + MMG	VML	F	*P* value
Defect weight (mg)	87.00 ± 6.73	-	79.75 ± 4.40	-	85.25 ± 5.46	87.38 ± 6.20	87.67 ± 3.18	93.11 ± 4.99	0.724	0.609

Asterisks represent differences by paired t-tests between limbs: * = *P* < 0.05.

Bold values indicates statistical significant *P* values (*P* < 0.05).

### Muscle weights are increased following bilateral VML injuries and MMG treatment

TA (wet) weights were not increased in limbs that received MMG treatment relative to untreated limbs (Main Effect, $\eta^{2}$ = 0.118, *P* = 0.093, ANOVA), but were increased in bilateral VML injured animals compared to unilateral VML injured animals (Main Effect, $\eta^{2}$ = 0.197, *P* = 0.004, ANOVA) (Table 1). The weights of the contralateral sham TAs were increased compared to the TA from the VML-involved limb in both the untreated (*P* < 0.001, paired t-test) and MMG-treated (*P* < 0.001, paired t-test) unilateral injury groups. TAs from untreated bilateral VML injured animals were equivalent in weight (*P* = 0.649, paired t-test), whereas the limb that received MMG was heavier than the untreated contralateral TA (*P* = 0.006, paired t-test) in the unilaterally MMG-treated bilateral VML injury group.

### Bilateral VML injuries do not exacerbate the systemic inflammatory response

**Fig. 1:**
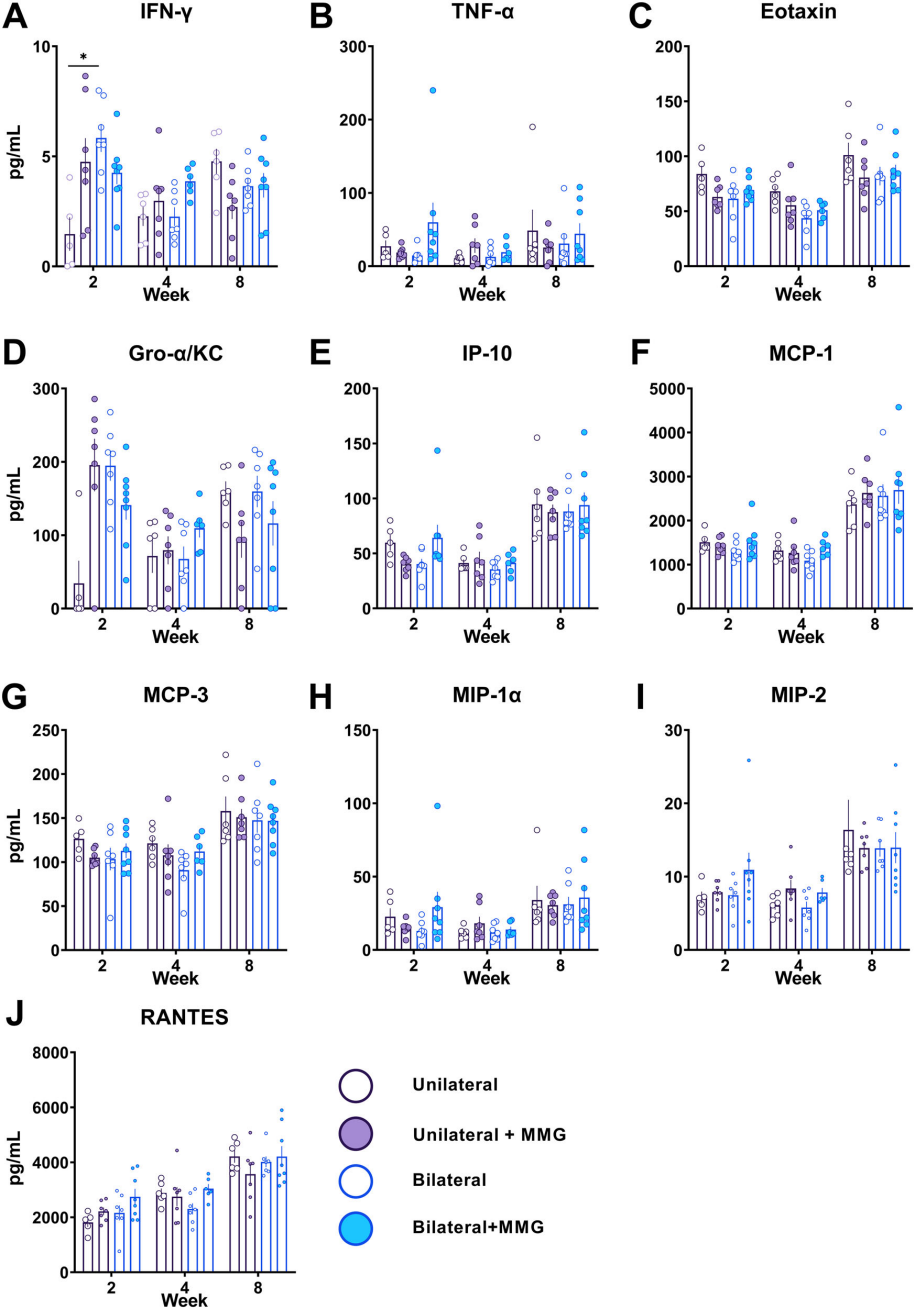
Changes in systemic chemokines and cytokines in bilateral and unilateral injured animals. Quantification of serum collected at 2, 4, and 8 weeks post-VML injury for **A** interferon-gamma (IFN-ℽ), **B** tumor necrosis factor-alpha (TNF-*ɑ*), **C** Eotaxin, **D** Gro-alpha/keratinocytes-derived chemokine (Gro-*ɑ*/KC), **E** Interferon gamma-induced protein 10 (IP-10), **F** monocyte chemoattractant protein-1 (MCP-1), **G** MCP-3, **H** macrophage inflammatory protein 1-alpha (MIP-1*ɑ*), **I** MIP-2, and **J** regulated on activation, normal T cell expressed and secreted (RANTES). Data is presented as mean ± SEM. * = *P* < 0.05.

Serum was collected at 2-, 4-, and 8-weeks post VML injury to determine if the presence of a bilateral VML injury increased systemic inflammation, compared to unilateral VML, and if MMG treatment exhibits a damping effect on systemic inflammation compared to no treatment. Ten of the possible 22 chemokines and cytokines reached our threshold for inclusion in analysis, and while 9 of 10 varied by time post- VML injury, only INF-γ (*P* < 0.001, ANOVA) and Gro-α/KC (*P* < 0.001, ANOVA) exhibited significant interactions between treatment and number of VML injuries that varied with time since injury **(**Fig. 1; Table 2). Multiple comparison tests show that INF-γ expression is upregulated in bilateral VML injured animals compared to unilateral VML injured animals at the 2-week time point (*P* = 0.042, Sidak’s Post hoc) but is similar at later time points (Fig. 1; Table 2). Of all three variables, time since injury had the greatest effect, with differences observed in 9 of 10 detectable analytes. Collectively, these data show that the number of injuries and treatment have minimal effect on the basal response to VML with respect to systemic chemokines and cytokines.

**Table 2:** Mixed models analysis of systemic chemokines and cytokines.

	IFN-γ	TNF-α	Eotaxin	Gro-α/KC	IP-10	MCP-1	MCP-3	MIP-1α	MIP2	RANTES
Fixed effects (type III)	*P* value									
Week	**0.025**	0.142	**<0.001**	**0.004**	**<0.001**	**<0.001**	**<0.0001**	**<0.001**	**<0.001**	**<0.001**
Treatment	0.363	0.395	0.290	0.573	0.693	0.224	0.859	0.441	0.415	0.272
# of injuries	**0.040**	0.715	0.071	0.068	0.887	0.947	0.276	0.911	0.944	0.320
Week x Treatment	**0.031**	0.482	0.743	**0.005**	0.906	0.857	0.687	0.887	0.229	0.133
Week x # of injuries	0.065	0.687	0.619	0.376	0.857	0.682	0.890	0.879	0.488	0.335
Treatment x # of injuries	0.390	0.143	**0.023**	0.061	0.051	0.401	0.134	0.209	0.400	0.073
Week x Treatment x # of injuries	**<0.001**	0.210	0.929	**<0.001**	0.189	0.570	0.429	0.236	0.703	0.570

Bold values indicates statistical significant *P* values (*P* < 0.05).

### Bilateral VML injuries exhibit reduced functional deficits relative to a unilateral VML injury

**Fig. 2:**
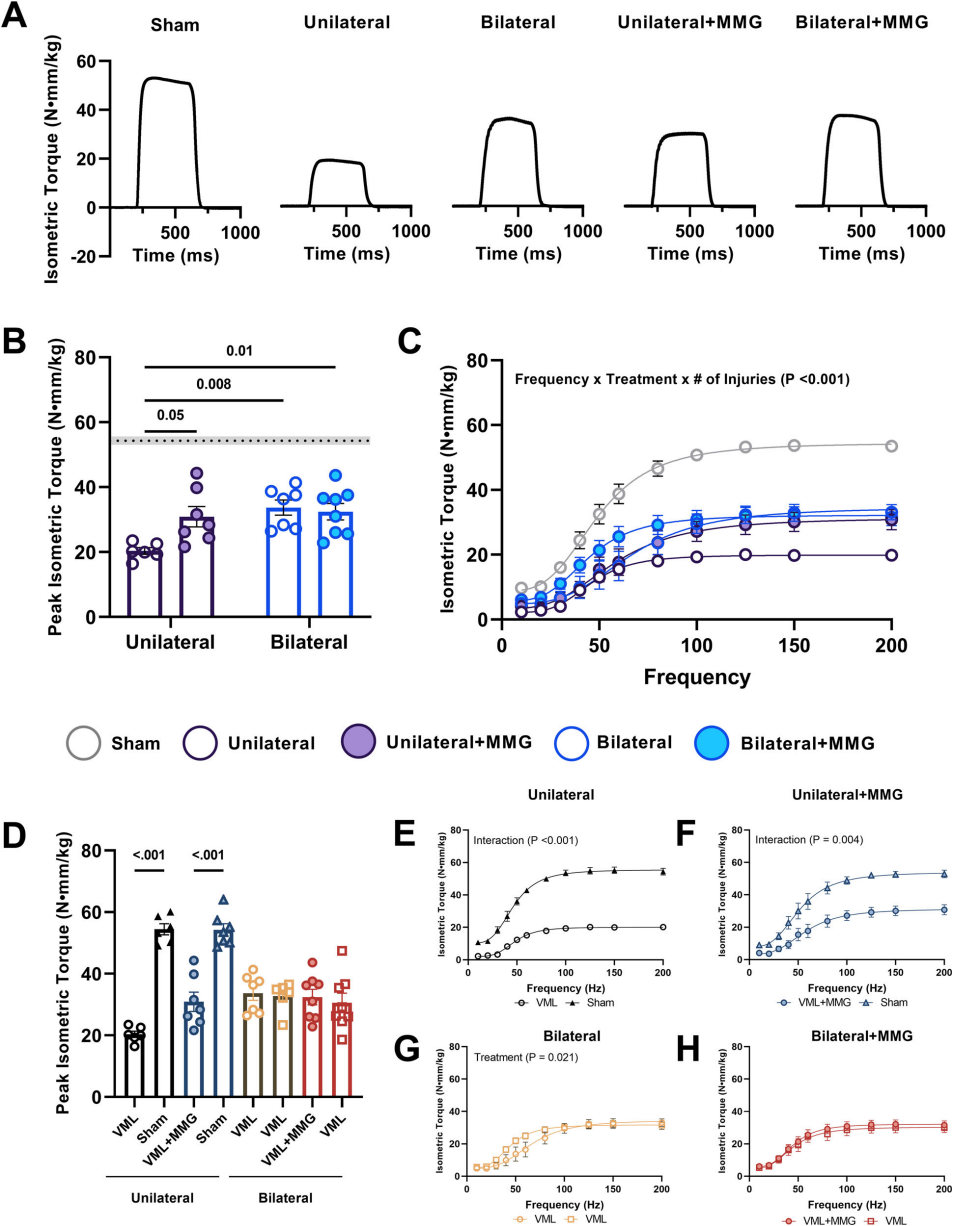
In vivo neuromuscular function of the TA muscle. Quantification of in vivo TA muscle neuromuscular strength was assessed at 8-weeks post injury. **A** Isometric torque tracings at 150 Hz, **B** peak isometric torque, and **C** isometric torque as a function of stimulation frequency are presented for the left hindlimb of each group and normalized to body weight. The dashed line and shaded region represent the average peak isometric torque of the contralateral limbs of the unilaterally injured groups. Additionally, peak isometric torque (**D**) and average maximal TA torque as a function of frequency (**E**–**H**) are presented for the right hindlimb alongside the limb of interest (i.e., left) and broken out by group. Data is presented as mean ± SEM. * = *P* < 0.05.

In accordance with the prior literature, peak isometric torque of untreated, unilateral VML injuries was reduced (*P* < 0.0001, paired t-test) relative to their contralateral sham controls (Fig. 2A, B). When bilateral VML injuries are considered alongside the traditional unilateral VML data, peak isometric torque of VML-affected TA muscles was found to be higher (Main Effect, ANOVA, $\eta^{2}$ = 0.220, *P* = 0.007) than their unilaterally injured counterparts (Fig. 2A, B). Specifically, we observed that for untreated VML injuries, peak isometric torque was 65.7% greater in the bilateral model relative to the unilateral model, and no difference in peak isometric torque was observed between limbs (*P* = 0.936, ANOVA) within the group with bilateral untreated VML injuries (Fig. 2B). When analyzing the force–frequency curves (Fig. 2C), isometric torque was likewise found to be increased in bilaterally VML injured animals relative to unilaterally VML injured animals across the range of frequencies investigated (Main Effect, ANOVA, $\eta^{2}$ = 0.045, *P* = 0.037).

### MMG mediated functional recovery is abrogated in animals with bilateral VML injury

In accordance with the prior literature, MMG treatment improved peak isometric torque by 51.9% (*P* = 0.049, Sidak’s Post hoc) over untreated injuries in a unilateral VML model (Fig. 2A, B). When these results are considered alongside data from the bilateral VML injury model, however, an interaction (ANOVA, $\eta^{2}$ = 0.138, *P* = 0.027) between the number of VML injuries and MMG treatment suggests the observed efficacy of MMG in the unilateral VML injury model is diminished in a bilateral VML injury model (Fig. 2A, B). Moreover, pairwise comparisons revealed the peak isometric torque for the MMG-treated muscles within the bilateral VML injury model was not different from their untreated contralateral injuries (*P* = 0.559, Sidak’s Post hoc) (Fig. 2B). Analysis of the torque–frequency curves, reveals a 3-way interaction between stimulation frequency, number of injuries, and treatment condition (ANOVA, $\eta^{2}$ = 0.016, *P* < 0.001) implying the observed interaction between number of VML injuries and treatment condition for tetanic contractions does not hold for all stimulation frequencies (Fig. 2C). Analysis of torque-frequency curves of both limbs (Fig. 2D–H) further illustrates the disparities in functional outcomes associated with MMG treatment in unilateral and bilateral models.

### MMG treatment increases PAX7 and decreases COL1A1 abundance in muscles with VML injuries

**Fig. 3:**
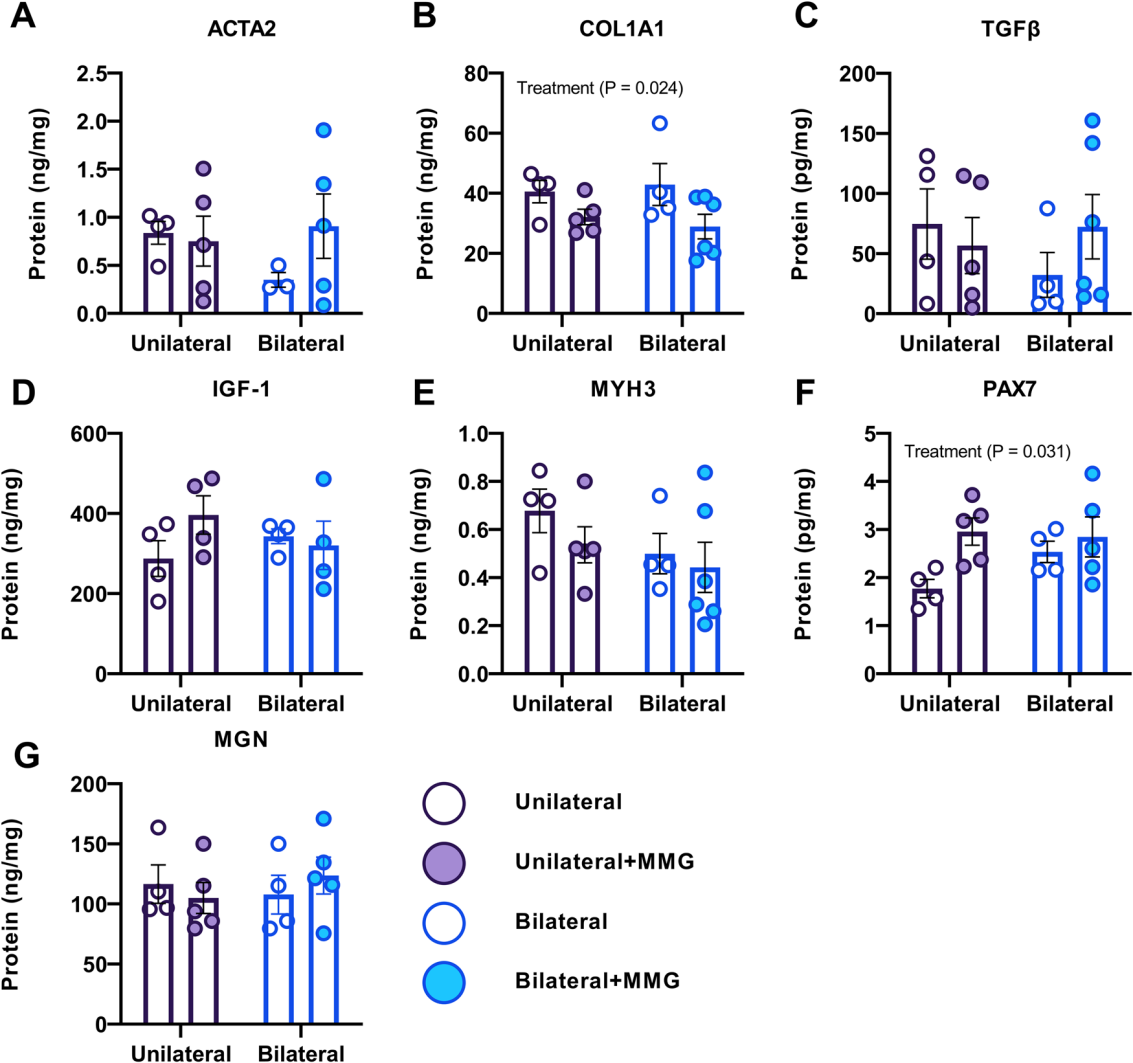
Protein analysis of bilateral and unilateral injured TAs, with or without, MMG treatment. Quantification of protein levels using ELISAs at 8 weeks post-VML injury for **A** alpha smooth muscle actin (ACTA2), **B** collagen type 1 alpha 1 (COL1A1), **C** transforming growth factor beta (TGFβ), **D** insulin-like growth factor (IGF), **E** myosin heavy chain 3 (MYH3), **F** PAX7, and **G** myogenin (MGN). Data is presented as mean ± SEM.

**Fig. 4:**
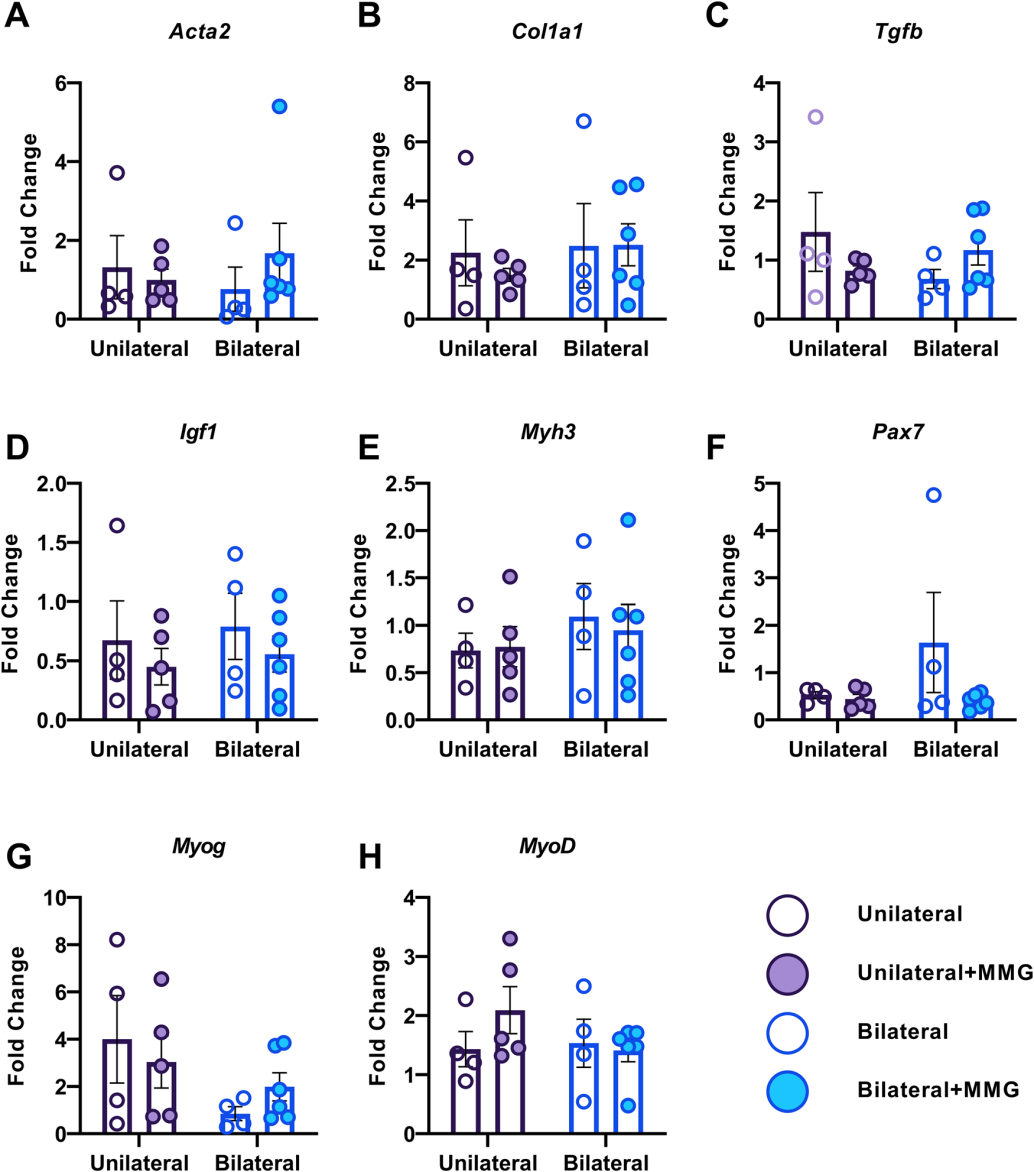
Transcriptional analysis of bilateral and unilateral injured TAs, with or without, MMG treatment. Quantification of transcription levels by RT-qPCR at 8 weeks post-VML injury for **A** alpha smooth muscle actin (Acta2), **B** collagen type 1 alpha 1 (Col1a1), **C** transforming growth factor beta (Tgfb), **D** insulin-like growth factor (Igf1), **E** myosin heavy chain 3 (Myh3), **F** Pax7, **G** myogenin (Myog), and **H** myoblast determination protein 1 (MyoD). Data is presented as mean ± SEM. * = *P* < 0.05.

Various markers implicated in myogenesis and fibrosis were evaluated within muscles harvested at 8-weeks post VML injury to determine the pathophysiology-based effect of the number of VML injuries on the compositional outcomes of VML treated with or without MMG. Interestingly, from both protein (Figs. 3 and S1) and gene (Figs. 4 and S2) analyses, we were unable to determine any biochemical differences at 8-weeks after injury between unilateral and bilateral VML-injured animals. Analysis of protein expression revealed MMG treatment (Main Effect, ANOVA, $\eta^{2}$ = 0.255, *P* = 0.031) resulted in increased abundance of PAX7, a transcription factor expressed in satellite cells involved in myogenesis, and decreased abundance of COL1A1, the major component of type I collagen and which is deposited during fibrosis (Main Effect, ANOVA, $\eta^{2}$ = 0.287, *P* = 0.024) relative to untreated injuries but did not alter myogenin, TGFβ, ACTA2, IGF-1, or MYH3 expression (Fig. 3). Moreover, multiple comparisons tests revealed unilateral and bilateral VML injured animals that received MMG-treatment had elevated PAX7 levels compared to the contralateral sham (*P* = 0.043, Sidak’s Post hoc) and untreated (*P* = 0.011, Sidak’s Post hoc) limbs, respectively (Fig. S1). Analysis of gene expression for *Myog, MyoD, TGFβ, Acta2, Col1a1, Pax7, Igf1, and Myh3* showed that there were no main effects or interactions (*P* > 0.05, ANOVA) between the independent variables (Figs. 4 and S2).

### Bilateral VML injury does not increase fibrosis compared to unilateral VML injury

**Fig. 5:**
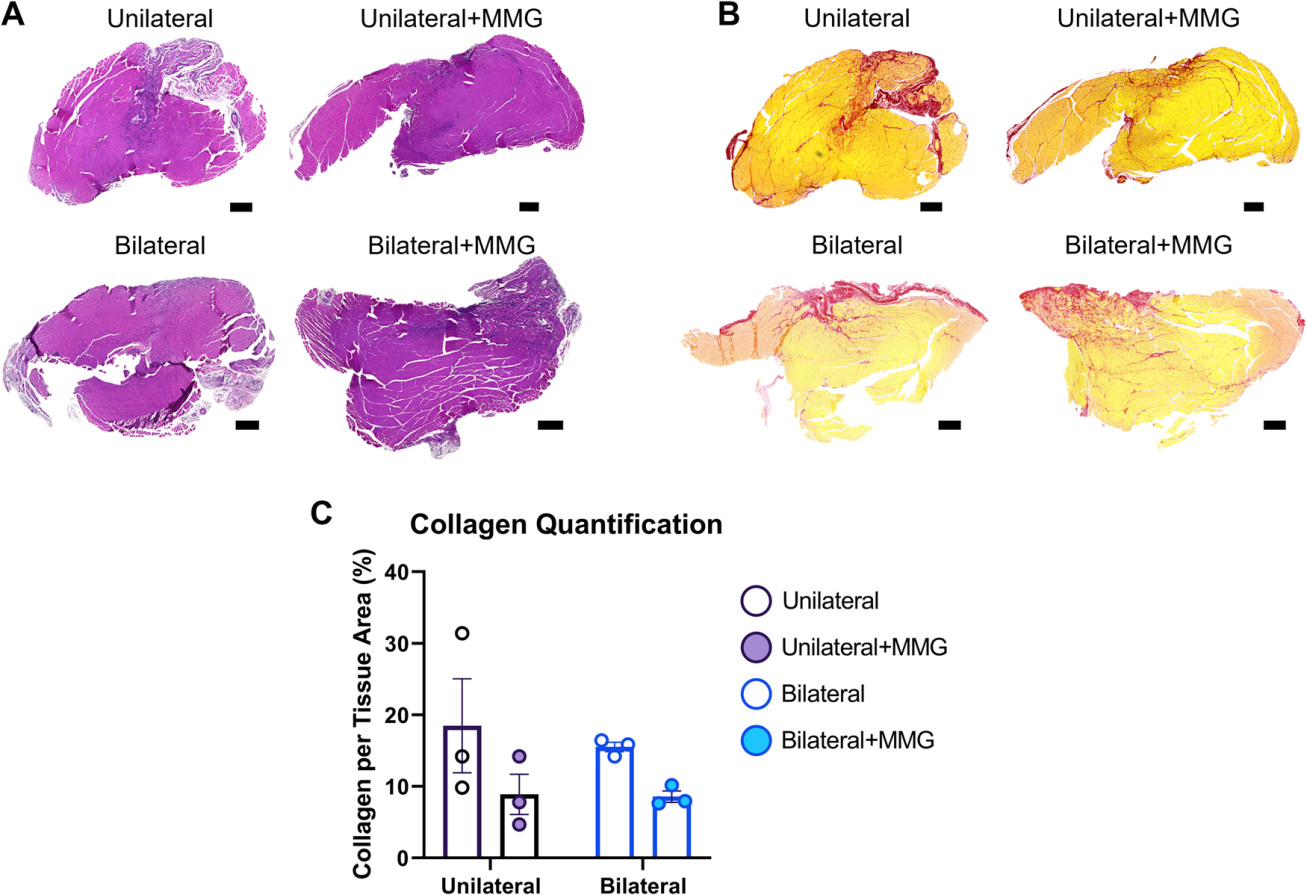
Histological analysis of unilateral and bilateral injured TA muscles. Histology of TA muscles at 8 week post-VML injury with **A** hematoxylin and eosin and **B** picrosirius red staining. Scale bars = 500 µm. **C** Quantification of collagen deposition in VML injured TA muscles.

Affected muscles were assessed for histopathological changes by staining muscle cross sections with hematoxylin & eosin staining (Fig. 5A) and picrosirius red (PSR) 8 weeks post-VML injury (Fig. 5B). No difference was observed with respect to fibrotic tissue deposition between unilateral and bilateral injured animals (Main Effect, ANOVA, $\eta^{2}$ = 0.015, *P* = 0.663) (Fig. 5B, C). Furthermore, there was also no statistically discernable difference in PSR staining owing to MMG treatment in this study (Main Effect, ANOVA, $\eta^{2}$ = 0.385, *P* = 0.051) (Fig. 5B, C).

## Discussion

Within a VML injury, the inflammatory response is prolonged and heightened, and well known to exacerbate fibrosis^15,36^. For instance, the complement pathway, TGF-β, and Wnt signaling are up-regulated weeks after VML injury and collectively promote fibrosis by attenuating satellite cell activity, increase fibroblast collagen synthesis, and down-regulate genes for myogenic transcription factors in rat and porcine models^15,31,36^. Based on the fact that the total injury burden is increased in a multiply injured scenario relative to single/isolated injuries, we hypothesized that bilateral VML injuries would exacerbate the systemic inflammation of unilateral VML and thus have a compounding effect on the known local immune dysregulation associated with such injuries. Our data, however, contradicts that hypothesis as bilateral VML injuries had minimal effect on the basal systemic inflammatory response to unilateral VML (Fig. 1). For instance, while we see differences in Eotaxin, IFN-γ, and GRO-α/KC at 2-weeks, the dynamics of most systemic chemokines/cytokines were similar between unilateral and bilateral VML injured animals. Furthermore, MMG treatment was not found to have a meaningful effect on systemic cytokine levels. These findings are concordant with the idea that a singular VML injury is sufficient to initiate the inflammatory cascade, but the modulatory impact of a second injury, or MMG treatment thereof, likely has a negligible impact on tissue level inflammation.

In addition to the counterintuitive finding that multiple VML injuries do not have a meaningful impact on the systemic inflammatory response above and beyond that of a single injury, we also found that bilateral VML injuries surprisingly exhibited better functional recovery compared to unilateral injuries (Fig. 2). As we did not observe any differences in myogenic markers nor fibrosis between untreated VML injuries from unilateral and bilateral injury models (Figs. 3, 4), we hypothesize that the increased torque production observed in the bilaterally VML injured animals may be mediated either through hypertrophy of existing myofibers and/or remodeling of the organization and structure of accumulated fibrous matrix for improved force transmission (i.e., functional fibrosis) possibly stemming from compensatory locomotion strategies. However, the data presented herein are insufficient to specifically test this hypothesis and thus further study is necessary to ascertain a precise mechanism underlying the observed functional phenomenon.

Another interesting finding from the current study is that the efficacy of MMG for mediating partial recovery of muscle force production after unilateral VML is abrogated in the bilateral VML model. While it is possible this observation represents a ceiling effect on functional recovery of VML injuries owing to other aspects of the pathology (e.g., architectural remodeling, metabolic dysfunction, denervation), it is plausible the inherent difference in unilateral and bilateral models can at least partially explain the observation. It is worth considering other studies that utilized a bilateral study design to evaluate functional outcomes after the application of an acute therapy. Interestingly, in bilateral rodent studies which show molecular and/or histological improvements with a direct acute therapy^14–26^, relatively few show improvements over the untreated condition via neural evoked neuromuscular functional assessments (in vivo or in situ) without an adjunct rehabilitative intervention initiated at a delayed time point^21,43^, an observation that is not as common in unilateral study designs. In porcine studies, results are somewhat mixed with some reports showing an effect of a therapeutic intervention^27^, whereas others do not show the same effect^30^, and yet others are uninterpretable from this standpoint given the incorporation of a sham control group into the random allocation of experimental conditions within their bilateral design^31^. An obvious challenge to our results presented herein, is the work by Ward et al.^27^, which showed MMG to be effective at promoting improved neuromuscular function in a bilateral pig model of VML. While we cannot fully explain why we do not observe a similar result in our small animal model, there are several anatomical and physiological differences that could ultimately account for these differences. Specifically, differences with respect to body weight, sex, metabolic profiles, general activity levels, and differences in plantigrade and unguligrade (e.g., typical range of motion, stride length, dorsiflexor recruitment) gait that could have non-trivial effects on the VML wound bed within the respective lower hindlimb dorsiflexor muscles of these disparate species.

Collectively, our results emphasize the need for future studies into the mechanisms of functional recovery after bilateral and unilateral VML injuries. Supporting this idea, our data demonstrate that bilateral VML injuries experience a similar systemic inflammatory response, but an improved muscle functional recovery compared to unilaterally injured animals. Moreover, we find that the efficacy of a regenerative therapeutic varies between unilateral and bilateral models. These results suggest that one should proceed with caution before comparing results between studies that differed in experimental design with respect to comparing unilateral to bilateral VML injury models, and emphasize the importance of further mechanistic investigations and preclinical model selection for early stage screening of putative therapies.

## Methods

### Experimental design

Adult male Lewis rats (366 ± 17 g, Charles River, Wilmington, MA) were maintained on a 12 h light-dark cycle in a vivarium accredited by the American Association for the Accreditation of Laboratory Animal Care with ad libitum access to food and water. Prior to surgery, rats were randomly assigned into one of four experimental groups: (1) unilateral VML injury, no treatment (*n* = 7), (2) bilateral VML injury, no treatment (*n* = 8), (3) unilateral VML injury treated with MMG (*n* = 8), and (4) bilateral VML injuries of which only one limb was treated with MMG (*n* = 9). At 8 weeks post-VML injury, in vivo neuromuscular functional capacity was assessed in all limbs across all animals, except those specifically allocated for histological analyses. Animals were subsequently euthanized with a lethal dose of Euthasol (Virbac AH, Inc., Westlake, TX, USA) delivered via intracardiac injection and the anterior crural muscles of the hindlimbs were harvested for molecular analysis. Markers of systemic inflammation were assessed in the serum at 2-, 4-, and 8-weeks post-VML injury. Investigators were blinded during surgical creation of the injury model, neuromuscular functional assessments, and tissue collection, but unblinded for all other analyses. All protocols and animal care guidelines were approved by the institutional animal care and use committee at the Uniformed Services University of the Health Sciences (USUHS; Bethesda, Maryland). All experiments were conducted in compliance with the Animal Welfare Act, the Implementing Animal Welfare Regulations, and in accordance with the principles of the Guide for the Care and Use of Laboratory Animals.

### VML injury and MMG-treatment

Animals were subjected to the well-established tibialis anterior (TA) muscle based VML injury model (i.e., 6 mm full thickness biopsy) as previously reported by our group^44–46^. For unilateral VML injuries (groups 1 & 3), injuries were created on the left hindlimb and a sham-operation was performed on the contralateral (i.e., right) hindlimb. For MMG-treated groups (groups 3 & 4), the tissue ablated by the creation of the VML defect was weighed, minced with surgical scissors in a sterile petri dish, and then immediately implanted back into the TA. Any bleeding was controlled with light pressure, and the wound was closed in layers with absorbable sutures. For bilaterally injured animals receiving unilateral MMG treatment (Group 4), the therapy was delivered to the left hindlimb.

### Serological analysis

Blood was collected from the ventral tail artery at 2-, 4-, and 8-weeks post-VML injury, centrifuged for 15 min at 1000 × *g*, and then serum was collected, flash frozen, and stored at −80 °C. Circulating cytokines granulocyte colony-stimulating factor (G-CSF), granulocyte-macrophage colony-stimulating factor (GM-CSF), interferon gamma (IFN-ℽ), interleukin 1 alpha (IL-1α), IL-1βa, IL-2, IL-4, IL-5, IL-6, IL-10, IL-12p70, IL-13, IL-17A, tumor necrosis factor alpha (TNFα) and chemokines eotaxin, chemokine (C-X-C motif) ligand 1(GROα/KC), interferon gamma-induced protein 10 (IP-10), monocyte chemoattractant protein 1 (MCP-1), MCP-3, macrophage inflammatory protein 1 alpha (MIP-1α), MIP-2, CLL5 (RANTES) were quantified via multiplex immunoassay (Cytokine & Chemokine 22-Plex Rat ProcartaPlex™ Panel, Invitrogen) following the manufacturer’s protocol using Luminex xMAP technology (Bio-rad, Bio-plex 200 system) to evaluate changes in the inflammatory response. Analytes were excluded from further analysis if less than 75% of the total analyzed samples were below the detection limit, or if less than 75% of the samples in one of the animal groups were below the detection limit. For analytes that reached these thresholds, samples below the detection limit were assigned a value of half the lower limit of quantitation.

### Muscle functional assessment and tissue collection

In vivo functional testing of TA muscles was performed at 8-weeks post-injury. Briefly, in vivo physiological properties of the TA muscle were measured in anesthetized rats (isoflurane 1.5–2.0%) using a dual-mode muscle lever system (Model 305C, Aurora Scientific, Inc). Subcutaneous needle electrodes were inserted on either side of the common peroneal nerve, and an optimal voltage was set with a series of tetanic contractions (150 Hz, 0.1 ms pulse width, 400 ms train). Then, a skin incision was made at the antero-lateral aspect of the ankle and the distal tendons of the extensor digitorum longus (EDL) and extensor hallucis longus muscles were isolated and severed. TA muscle isometric tetanic torque was measured (10–200 Hz) with the ankle constrained at a right angle. This procedure was then repeated on the contralateral limb. For one animal in the bilateral VML injury, no treatment group, we were unable to measure torque in the contralateral limb because the TA tendon was accidentally severed. After all measurements were collected, the TA and EDL muscles were collected from each hindlimb, blotted, and weighed (wet).

### Protein extraction, RNA extraction, and quantification

Flash-frozen whole TA muscles were homogenized and half of the sample was allotted for protein extraction, the other half for RNA extraction. For protein extraction, the homogenized tissue was lysed in T-PER tissue protein extraction reagent (Catalog# 78510, Thermo Scientific) with Halt protease inhibitor cocktail (Catalog# 78429, Thermo Scientific). Diluted samples were then assessed via enzyme-linked immunosorbent assay (ELISA) for insulin growth factor-1 (IGF1) (Catalog# MBS268050, MyBiosource, Inc.), myogenin (Catalog# MBS9712815, MyBiosource, Inc.), transforming growth factor beta (TGFβ) (Catalog# MBS824788, MyBiosource, Inc.), alpha smooth muscle actin (ACTA2) (Catalog# MBS266620, MyBiosource, Inc.), myosin heavy chain 3 (MYH3) (Catalog# MBS068782, MyBiosource, Inc.), collagen type 1 alpha 1 (COL1A1) (Catalog# MBS042769, MyBiosource, Inc.), and PAX7 (Catalog# MBS2606155, MyBiosource, Inc.) following the manufacturer’s protocols. Obtained protein values were normalized to total protein content as determined by Pierce™ BCA protein assay kit (Catalog# 23225, Thermo Scientific).

RNA was extracted from homogenized whole TA muscles using TRIzol reagent (Catalog# 15596026, Invitrogen) following the manufacturer’s protocol. Reverse transcription was then performed using Iscript Select cDNA Synthesis Kit (Catalog# 1708897, Bio-Rad) using random hexamer primers following the manufacturer’s protocol. The resulting cDNA then underwent RT-qPCR on a QuantStudio 7 Flex using iTaq Universal SYBR Green Supermix (Catalog# 1725122, Bio-Rad). Theoretical starting transcript concentrations were calculated using LinRegPCR based on individual PCR efficiency values (Table S1). Concentrations were then normalized to the geometric mean of values obtained for reference genes (18S rRNA, GAPDH).

### Picrosirius red staining, imaging, and quantification

Whole TA muscles (*n* = 3 samples per group) collected during tissue harvest were stored in 10% formalin (Sigma- Aldrich; St. Louis, MO, USA) for 1 week and then processed and embedded into paraffin. Tissues were then sectioned at a thickness of 6 μm and placed onto slides using a water bath. Slides were baked at 60 °C for 45 min, deparaffinized, rehydrated, and stained with either Hematoxylin and Eosin (MilliporeSigma; Burlington, MA) or Picrosirius Red (PSR; Abcam; Cambridge, United Kingdom) using the manufacturer protocols. Slides were sealed with Permount Mounting Medium, and then imaged at 20× magnification with an Axio Scan Z1 (Zeiss; Oberkochen, Germany) slide scanning microscope. For PSR-stained slides, image acquisition parameters were standardized for all samples being analyzed. For each TA muscle (*n* = 3/group), 3 cross-sections within the VML injury were imaged, quantified, and then averaged together prior to statistical analysis. Collagen quantification was conducted in ImageJ^44^, and restricted to the middle of the TA muscle where the VML injury was performed.

### Statistics

Data was analyzed using GraphPad Prism (GraphPad Software, 9.1.0). Dependent variables were analyzed using analysis of variance (ANOVA) or paired t-tests. In the event of a significant ANOVA, a Fisher’s or Sidak’s multiple comparison tests were performed. Statistical significance was achieved at alpha of 0.05.

### Reporting summary

Further information on research design is available in the Nature Research Reporting Summary linked to this article.

## Supplementary information

Supplemental Material

REPORTING SUMMARY

## Data Availability

All data generated or analyzed during this study are included in this published article (and its supplementary information files).
