# Supplementary material for: The impact of bilateral injuries on the pathophysiology and functional outcomes of volumetric muscle loss
Source: NPJ Regen Med. 2022 Oct 15;7:59. doi: 10.1038/s41536-022-00255-2 (PMC9569363; doi:10.1038/s41536-022-00255-2)
Supplement: Supplementary file 1 — Supplemental Material [file 41536_2022_255_MOESM1_ESM.pdf]

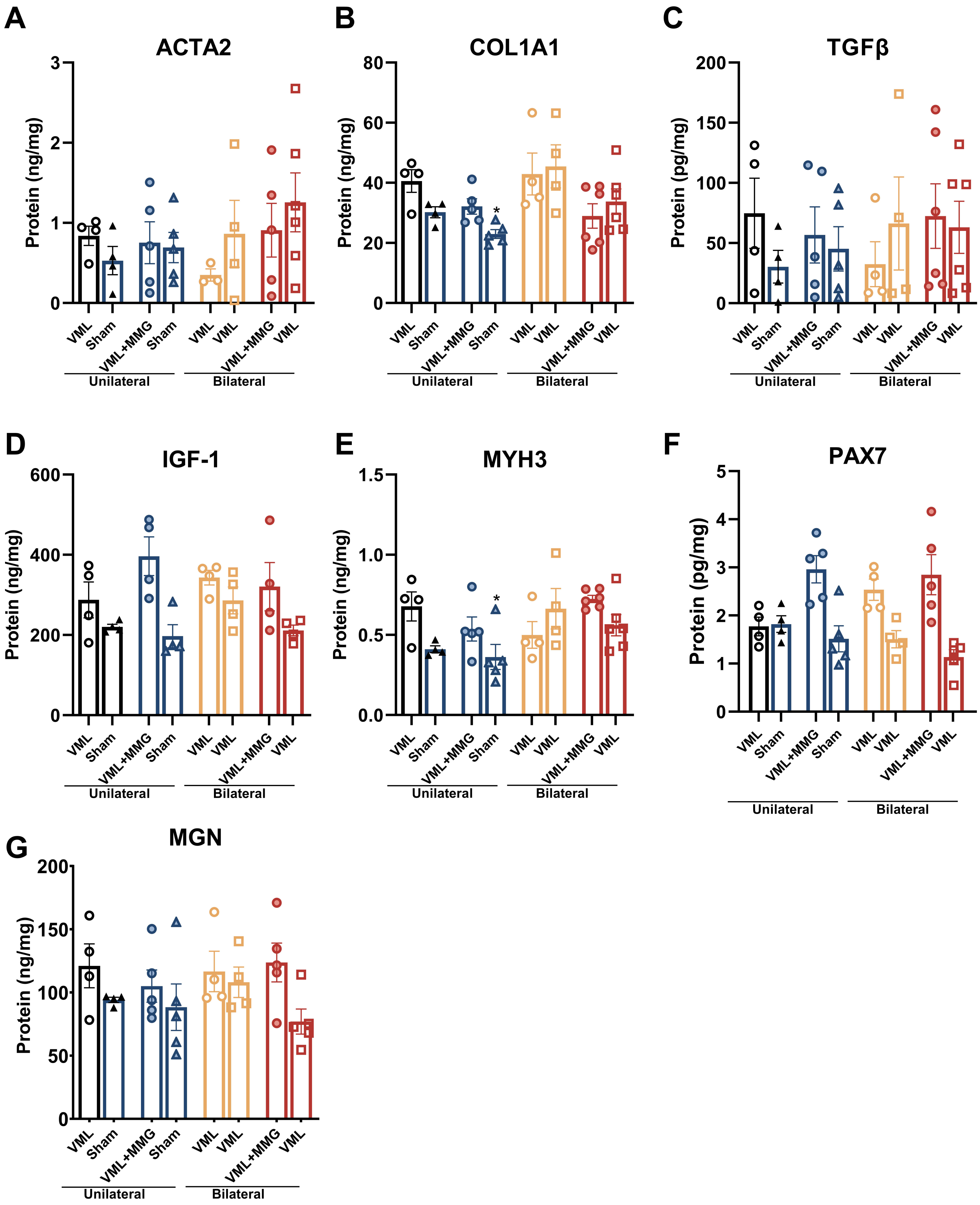
A
ACTA2
Protein (ng/mg)
B
COL1A1
Protein (ng/mg)
C
TGFβ
Protein (pg/mg)
D
IGF-1
Protein (ng/mg)
E
MYH3
Protein (ng/mg)
F
PAX7
Protein (pg/mg)
G
MGN
Protein (ng/mg)
VML
Sham
VML+MMG
Sham
VML
VML
VML+MMG
VML
Unilateral
Bilateral
*

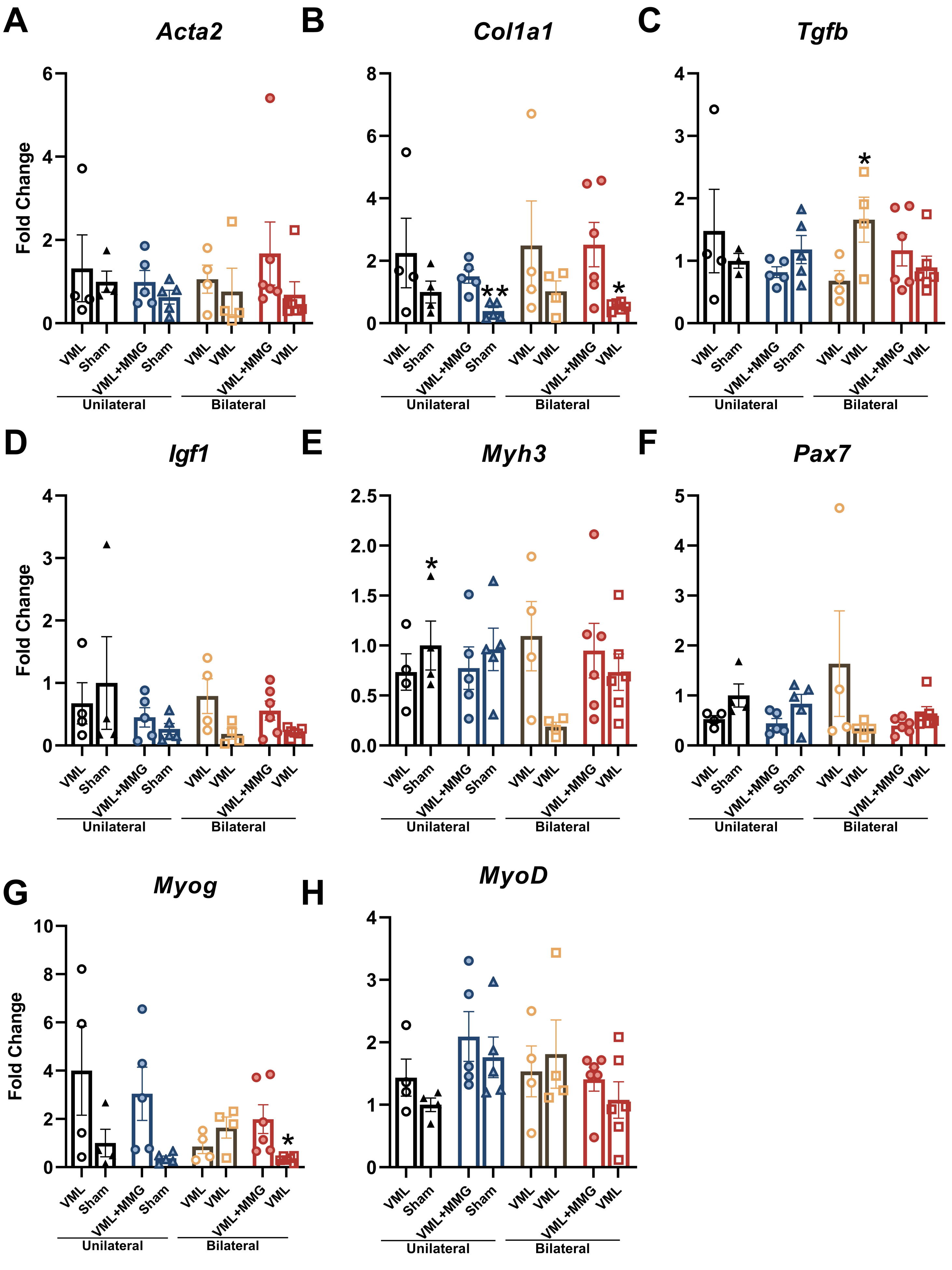

A
Acta2
B
Col1a1
C
Tgfb
D
Igf1
E
Myh3
F
Pax7
G
Myog
H
MyoD
Fold Change
VML
Sham
VML+MMG
Sham
VML
VML
VML+MMG
VML
Unilateral
Bilateral
**
*

Table S1: Primer Sequences for RT-qPCR

Transcript	Strand	Sequence	Accession Number
18S	Forward	5'- GGAGGTAGTGACGAAAAATAACAAT -3'	NR_046237.2
	Reverse	5'- TTGCCCTCCAATGGATCCT -3'	
Acta2	Forward	5'- AAACCACCTATAACAGCATC -3'	NM_031004.2
	Reverse	5'- AGACAGAATATTTGCCGTTCTG -3'	
Col1a1	Forward	5'- TGGATTCCAGTTCGTATG -3'	NM_053304.1
	Reverse	5'- AGTGATAGGTGATGTTCTGG -3'	
GAPDH	Forward	5'- AAGTTCAACGGCACAGTCAAGG -3'	NM_017008.4
	Reverse	5'- CATACTCAGCACCAGCATCACC -3'	
MGN	Forward	5'- TCCCAACCCAGGAGGTAAGT -3'	NM_017115.3
	Reverse	5'- CGCTGGAAGGTAACTGGAAC -3'	
MyoD	Forward	5'- TCGGCTACATTGAAGGTC -3'	NM_176079.2
	Reverse	5'- GTAATCCATCATGCCATCAG -3;	
TGFB	Forward	5'- GGAAATCAATGGGATCAGTC -3'	NM_021578.2
	Reverse	5'- CTGAAGCAGTAGTTGGTAAT -3'	
MYH3	Forward	5′- TGGAGGACCAAATATGAGACG -3′	NM_012604.1
	Reverse	5′- CACCATCAAGTCCTCCACCT -3′	

18S (18S Ribosomal RNA); Acta2 (Actin Alpha 2, Smooth Muscle); Arg1 (Arginase 1); Col1a1 (Collagen Type I Alpha 1 Chain); GAPDH (Glyceraldehyde 3-phosphate Dehydrogenase); MGN (Myogenin); MyoD (Myogenic Differentiation 1); TGFB (Transforming Growth Factor Beta 1); MYH3 (Myosin Heavy Chain 3)

Supplementary Figure 1: Intra-animal limb comparisons of protein markers of myogenesis and fibrosis. Comparison of protein differences between different limbs in the same animal by experimental group at 8 weeks post-VML injury for (A) alpha smooth muscle actin (ACTA2), (B) collagen type 1 alpha 1 (COL1A1), (C) transforming growth factor beta (TGFβ), (D) insulin-like growth factor (IGF), (E) myosin heavy chain 3 (MYH3), (F) PAX7, and (G) myogenin (MGN). Data is presented as mean ± SEM. * = $P<0.05$.

Supplementary Figure 2: Intra-animal limb comparisons of transcriptional markers of myogenesis and fibrosis. Comparison of transcriptional differences between different limbs in the same animal by experimental group at 8 weeks post-VML injury for (A) alpha smooth muscle actin (Acta2), (B) collagen type 1 alpha 1 (Col1a1), (C) transforming growth factor beta (Tgfb), (D) insulin-like growth factor (Igf1), (E) myosin heavy chain 3 (Myh3), (F) Pax7, (G) myogenin (Myog), and (H) myoblast determination protein 1 (MyoD). Data is presented as mean ± SEM. * = $P<0.05$.
